# The Progress in Reconstruction of Mandibular Defect Caused by Osteoradionecrosis

**DOI:** 10.1155/2023/1440889

**Published:** 2023-03-15

**Authors:** Nan Huang, Peihan Wang, Ping Gong, Bo Huang

**Affiliations:** ^1^West China School of Stomatology and National Center of Stomatology, Sichuan University, Chengdu 610041, China; ^2^State Key Laboratory of Oral Diseases and General Dentistry, West China Hospital of Stomatology, Sichuan University, Chengdu 610041, China

## Abstract

Osteoradionecrosis (ORN) is described as a disease with exposed, nonviable bone that fails to heal spontaneously or by means of conservative treatment after radiotherapy in at least 3 months. Though traditional theories in the early stage including hypoxic-hypocellular-hypovascular and fibro-atrophic in addition to new findings such as ferroptosis were put forward to explain the mechanisms of the osteoradionecrosis, the etiology of ORN is still unclear. With the high rate of occurrence in the head and neck area, especially in the mandible, this disease can disrupt the shape and function of the irradiated area, leading to a clinical presentation ranging from stable small areas of asymptomatic exposed bone to severe progressive necrosis. In severe cases, patients may experience pain, xerostomia, dysphagia, facial fistulas, and even a jaw defect. Consequently, sequence therapy and sometimes extensive surgery and reconstructions are needed to manage these sequelae. Treatment options may include pain medication, antibiotics, the removal of sequesters, hyperbaric oxygen therapy, segmental resection of the mandible, and free flap reconstruction. Microanastomosed free-flaps are considered to be promising choice for ORN reconstruction in recent researches, and new methods including three-dimensional (3-D) printing, pentoxifylline, and amifostine are used nowadays in trying increase the success rates and improve quality of the reconstruction. This review summarizes the main research progress in osteoradionecrosis and reconstruction treatment of osteoradionecrosis with mandibular defect.

## 1. Introduction

Radiotherapy in the perioperative period is frequently scheduled for patients with head and neck cancer (HNC). Osteoradionecrosis (ORN) is a serious late complication of radiotherapy (RT) for head and neck cancer, which exhibits as exposed, nonviable bone in an irradiated field, failing to heal spontaneously or by means of conservative treatment in at least 3 months after radiotherapy while being unrelated to tumor recurrence [1–3]. The mandible, playing an important role in facial esthetics and the function of the stomatognathic system, is a predisposed place for ORN [4]. The incidence of ORN at 5 years has been reported to range between 2% and 40% [4–6]. Despite the use of advanced preventive methods, the rate of mandibular osteoradionecrosis after radiotherapy reached 5% [7]. Mandibular osteoradionecrosis clinically presents itself as painful and denuded bone with or without purulent drainage and/or possible fistula formation [8, 9], and patients with mandibular osteoradionecrosis may suffer from pain, xerostomia, dysphagia, difficulty of mouth opening, ulcers, and facial fistulas [10, 11]. Therapeutic options may range from pain medication, antibiotics, the removal of sequesters, hyperbaric oxygen therapy, segmental resection, and free flap reconstruction [12].

Though destroying the integrity of the mandible and decreasing the quality of life in affected individuals, mandibular segmental resection is the mainstay for severe osteoradionecrosis [13, 14]. When serious symptoms such as pathological fracture or fistula occur, mandibular segmental resection combined with free tissue transfer is often the curative option [14]. However, some studies have demonstrated a significantly high risk of complications in reconstructing the mandible after osteoradionecrosis because of the problematic process of healing has resulted from radiation damage [14–17]. It is urgent to have a comprehensive command of new approaches to reconstruct the mandible with good management of complications. In this review, we will discuss the progress in ORN and the reconstruction of the mandible caused by osteoradionecrosis.

## 2. Progress of Osteoradionecrosis (ORN)

### 2.1. The Possible Mechanisms of ORN

Marx's hypoxic-hypocellular-hypovascular theory and Delanian's fibro-atrophic theory are the most widely accepted traditional theories about the mechanisms of ORN [18–20]. Marx [20] proposed his theory in 1980s, stating that radiation induced an endarteritis that resulted in tissue hypoxia, hypocellularity, and hypovascularity, which leaded to nonhealing wounds. The fibro-atrophic theory proposed by Delanian and Lefaix [19] suggested that radiation not only depletes the fibroblast populations, but also decreases fibroblasts' ability to produce collagen and the pathophysiological sequence is: free radical formation caused by radiation, endothelial dysfunction, inflammation, microvascular thrombosis formation, fibrosis and remodeling, and bone necrosis. Radiation induces cell death by various modes, including apoptosis, necrosis, autophagy, and mitoticcatastrophe [21]. Zhuang and Zhou [22] demonstrated that after radiation, gingival fibroblasts secrete exosomes which inhibit the osteogenic differentiation of human bone mesenchymal stem cells (hBMSCs). Furthermore, Støre et al. [23] stated that mixed bacterial or fungal infection may play a fundamental role in the pathogenesis of ORN and teeth can provide the entrance for microorganisms.

**Figure 1:**
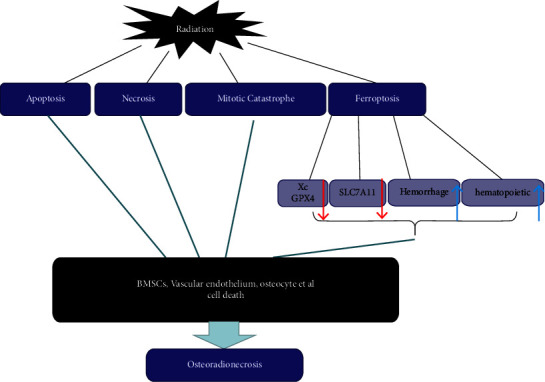
The possible mechanisms of ORN.

“Iron death” is a newly discovered form of cell death in recent years. Distinct from apoptosis, it is a phospholipid-peroxidation-driven, iron-dependent form of regulated cell death, which is theorized to contribute to many biological and diseases processes [24, 25]. Ye et al. [26] found genetic and biochemical hallmarks of ferroptosis in radiation-treated cancer cells and proposed that radiation could induce the inhibition of system Xc and GPX4 which would lead to ferroptosis. Lei et al.'s [27] work in 2020 stated that original radioresistant cancer cells become radio-sensitive by inactivating ferroptosis inhibitors SLC7A11 or GPX4. Zhang et al. [28] reported that radiation-induced hemorrhage in the bone marrow could result in ferroptosis, and antiferroptosis therapy may ameliorate the radiation-induced hematopoietic injury. These studies indicated that enhancing ferroptosis can make cancer cells more sensitive to radiotherapy. Therefore, a hypothesis can be made that ferroptosis may play a fundamental part in the development of ORN (Figure 1).

### 2.2. The Classification of ORN

**Figure 2:**
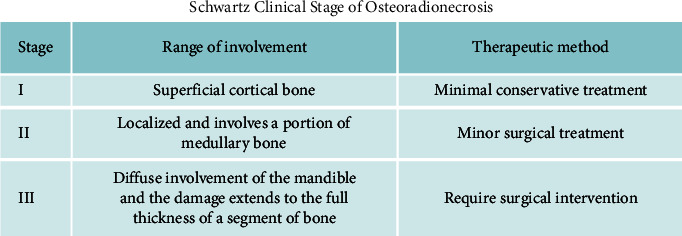
Schwartz clinical stage of ORN based on the extent of the necrosis.

In 2002, Schwartz and Kagan [29] developed a staging system for ORN from their 25 years of experience. His system is based on the extent of the necrosis. Superficial cortical bone involvement is stage I, and minimal conservative treatment is qualified. When the bone necrosis is localized and involves a portion of the medullary bone, it is classified as stage II, and minor surgical treatment may be required. When there is diffuse involvement of the mandible and the damage extends to the full thickness of a segment of bone, it is called stage III. All cases in stage III require surgical intervention (Figure 2) This classification was considered reliable in the 14^th^ Chinese Academic Conference on Oral and Maxillofacial Surgery [30].

**Figure 3:**
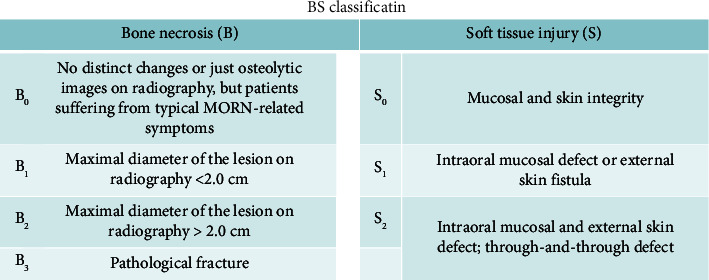
Bone and soft tissue (“BS”) staging system for mandibular osteoradionecrosis.

**Figure 4:**
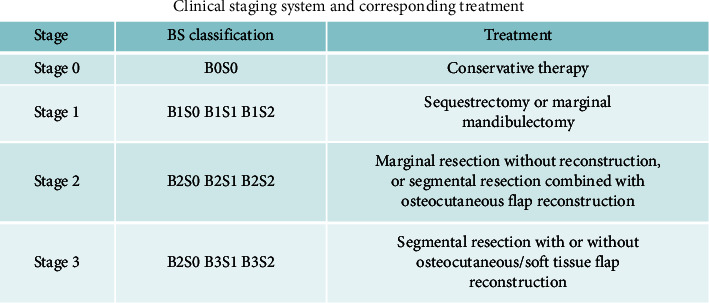
Treatment protocols according to the BS staging system.

Combined with both the presentation of disease and radiological findings, the Chinese Society of Oral and Maxillofacial Surgery (CSOMS) established a new classification for mandibular osteoradionecrosis in 2014 [31]. This system focuses on both hard and soft tissues, so it is also called the bone and soft tissue (“BS”) staging system. The B system determines the stage, while the S system represents the severity of the soft tissue injury in the same stage. From B_0_ to B_3_, the severity of hard tissue lesions increases and the S staging system goes the same way. Patients are divided into four and three stages, respectively, in the B (0–3) and S (0–2) systems. And the treatment protocols are designed according to the BS staging system. Conservative treatments are always given to stage 0 patients, while surgical treatments are limited to stages I, II, and III (Figures 3 and 4).

Different classification methods have different advantages and disadvantages for the development, recognition, and treatment of ORN. However, no stage remains a stationary condition, and the further course of disease progression is mainly unpredictable, making it a moving target. The ORN classification needs to be adjusted and the treatment methods need to improve according to the changes in the disease to achieve the best treatment effect.

## 3. Reconstruction of an ORN-Caused Mandibular Segmental Defect with Loss of Continuity

### 3.1. Flaps Used in the Reconstruction of an ORN-Caused Mandibular Defect

**Figure 5:**
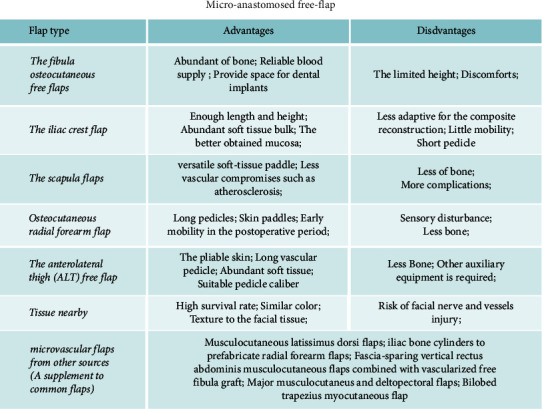
The characteristics of flaps used in the reconstruction of an ORN-caused mandibular defect.

For extended mandibular defects induced by osteoradionecrosis, microanastomosed free-flap combined with reconstructive plates or autogenous bone was considered to be a promising choice recently [32–35]. Traditionally, the flaps are harvested from fibular, iliac crest, scapular, and radial forearm [36–38]. These flaps have different features, which should be considered before clinical use (Figure 5).

The fibula osteocutaneous free flaps are acknowledged as one of the most common methods used for mandibular reconstruction [35, 39]. It can provide a long pedicle with a pliable skin paddle (10–20 cm), abundant bone stock (up to 25 cm), and reliable blood supply despite several osteotomies. It can be applied as a vascular runoff and a second free flap [14, 33, 40, 41]. Its bicorticocancellous structure makes the placement of dental implants to improve patients' quality of life feasible, and the donor site of it allows two teams to work at the same time, reducing the operation time and patients' sufferings [14]. However, one of the major problems with the fibula flap is the limited height (about 15 mm) in mandible reconstruction, and it may lead to longer abutments for implants, inappropriate crown to implant ratio and eventually the increased implant failure rate [41, 42]. In addition, fibula harvesting is usually associated with ankle instability and valgus deformity, which bring patients great discomforts [43]. What's more, the fibula osteocutaneous free flap is not suitable to every case, too, because suitable donor site is offen limited for vascular compromises such as venous thrombosis and atherosclerosis, previous trauma or surgery, extensive and complex soft tissue defects and unfavorable anatomy or dimensions [35, 43–46].

Compared to the fibula flaps, the iliac crest flap provides less width but enough (up to 15 cm) length of bone tissue for mandibular reconstruction, and higher bone height can be obtained to allow the use of longer implants. The vascularized iliac crest flaps also have abundant soft tissue bulk for intraoral soft tissue reconstruction, and the final texture of the obtained mucosa is better than other kinds of flaps [39, 47]. However, the free iliac crest flap is not very adaptive for the composite reconstruction, because its skin paddle is supported by deep circumflex iliac artery with little mobility and short pedicle, which leads to limited flexibility [48]. Moreover, donor-site morbidity may be significant.

The amount of bone offered by the scapula flaps, which are less popular, is limited compared to the iliac crest flaps, but they have their own advantages. The scapula flaps can provide versatile soft-tissue paddling up to 11-12 cm for large, complex defects [41]. When the reconstruction requires large amounts of soft tissue or the tongue and the soft palate and facial skin is involved, the scapula flaps are often recommended [35, 49]. Moreover, scapula flaps have less vascular compromises such as atherosclerosis than lower extremity vessels, which ensures unimpeded ambulation after the operation [50–52]. However, complications such as functional morbidity of the shoulders should be taken into consideration [53].

The anterolateral thigh (ALT) free flap is an ideal option for soft-tissue reconstruction of the head and neck, too [54]. Anterolateral thigh free flaps were combined with reconstruction plates by C. Bowe to reconstruct lateral posterior segmental mandibular defects [35]. Tursun et al. [55] also conducted a retrospective case series study which revealed that, combined with tensor fasciae latae flaps, anterolateral thigh free flaps can reconstruct large soft tissue defects (greater than 20 cm × 10 cm) in head and neck. The advantages of ALT flaps include the pliable skin, long vascular pedicle, abundant soft tissue and suitable pedicle caliber it offers, the minimal donor site morbidity and the usage of two team approach [54, 55]. Tissue nearby the defects can also be taken into consideration when harvesting the flaps. Due to submental flap's easy harvesting, high survival rate and similar color and texture to the facial tissue [56], Li et al. [57] combined it with reconstruction titanium plate to treat mandibular osteoradionecrosis, and out of 23 patients, only 1 failed due to partial necrosis in the distal end. He suggested that only when the flap is about 10% larger than the defect can this flap be used to ensure the wound closing without tension, and mandibular branch of the facial nerve and the vessels should be protected during the surgery.

Horta et al. [58] recently described the use of the facial artery perforator flap for osteoradionecrosis intraoral reconstruction. Though one of the patients had minor flap loss and dehiscence, no local recurrences were observed, and functional outcomes were satisfactory, so these cases were considered successes. Woo [59] reported a case treated with buccinator myo-mucosal flap. The flap and the buccal fat were used to fill the defect site, and the buccal mucosa was sutured to the defect site's mucosa. After a year's worth of observation, both the donor site and recipient site were considered healed. Only a linear scar remained at the donor site, and no contracture occurred. The osteocutaneous radial forearm flap provides long pedicles and skin paddles and is often used to promote early mobility in the postoperative period [60].

Combined with bone grafts, microvascular flaps from other sources can be used in ORN reconstruction, too. Hillerup et al. [46] harvested musculocutaneous latissimus dorsi flaps and wrapped the muscle fans of them around the fixed reconstruction plates in the mandibles to reconstruct soft tissues. After 3 to 6 months, particulate iliac bone grafts were inserted into the well-vascularised soft tissues provided by the flaps to complete the reconstruction. Leonhardt et al. [61] harvested iliac bone cylinders to prefabricate radial forearm flaps and later used these prefabricated flaps to successfully reconstruct 5 cases of mandibular defects. Kenney and Kiil [62] reported 4 cases using fascia-sparing vertical rectus abdominis musculocutaneous flaps combined with a vascularized-free fibula graft to reconstruct advanced mandibular osteoradionecrosis. This year, pectoralis major musculocutaneus and deltopectoral flaps, as well as bilobed trapezius myocutaneous flaps, were reported to be implemented in the reconstruction of the irradiated mandible[63, 64]. When common flaps such as fibular flaps and iliac crest flaps are not available, these flaps may bring new options to choose from.

### 3.2. New Progress in the Reconstruction of the Mandibular Defect Caused by ORN

#### 3.2.1. Use of Pentoxifylline Combined with Vitamin E

In recent years, pentoxifylline combined with vitamin E has been used clinically to treat osteoradionecrosis. The main mechanism is to inhibit fibrosis induced by radiation. And in 2023, Assim Banjar reviewed the articles published between 1997 and 2020 on the treatment of ORN with Pentoxifyline and vitamin E and found that the early use of Pentoxifyline and vitamin E could significantly reduce the severity of ORN and reduce the degree of injury [65–67]. It is mainly because Pentoxifyline and vitamin E could effectively inhibit the production of free oxygen, thus reducing the damage caused by radiation to bones. And they can inhibit the production of TGF-*β*, collagen, and fibronectin. The formation of tissue fibrosis caused by radiation and the occurrence of reflective osteonecrosis could be reduced [68–70]. Although the study found that the occurrence of radiation-induced jaw necrosis could not be completely inhibited, it could significantly improve the quality of life of patients, improve the opening degree of patients in the late stage of radiotherapy, and reduce the damage to salivary glands and jaws [71, 72].

#### 3.2.2. Use of Amifostine

Radiation protectors, with the goal of preferentially radio-protecting normal tissue while radio-sensitizing tumor cells, are a class of agents designed to reduce the cytotoxic effects of radiation. Amifostine (WR-2721), as the first and only radiation protector with authority from the Federal Food and Drug Administration (FDA) of the United States of America is widely used clinically to protect the normal cells of patients undergoing radiotherapy or chemotherapy has proven effective in reducing xerostomia in patients receiving postoperative head and neck radiation therapy [73–76]. Therapeutic radiation generally causes most of its damage through the production of reactive oxygen species (ROS), and the main target for these ROS is the cell's DNA. Damage caused by free radicals to the DNA can result in double or single strand breaks, leading to disruption of cell viability and culminating in cell death or senescence [74]. Amifostine is a phosphorothionate that works through its active thiol metabolite WR-1065 by free radical scavenging [77]. WR-2721 will only be taken up by cells when dephosphorylated to WR-1065, and within the cell, the WR-1065 can be oxidized further into WR-33278. Often, Amifostine will have a higher concentration in normal cells than in neoplastic cells, thus a higher portion of WR-33278 oxidized from WR-1065 may be attained to cause DNA condensation, reducing the area for free radical attack [78]. Apart from the views above, some evidence also shows that WR-1065 supports DNA repair in normal cells and inhibits DNA repair in cancer cells [79–81]. Bone marrow suppression is one of the most prominent effects of radiation-induced injury, Yu et al. [82] developed an amifostine-loaded armored microneedle (AAMN) with transdermal delivery system of amifostine for long-term protection against ionizing radiation-induced injury. The results suggested that the AAMN with deep skin insertion and high drug permeation can protect the hematopoietic stem cells and progenitor cells in bone marrow by effectively reducing the radiation-induced damage. The drug release in AAMN group was 3–7 h administration preradiation while merely 0.5-h in amifostine injection group. King et al. [74] reviewed 21 randomized, controlled, prospective trials in head and neck cancer and concluded that amifostine may reduce the incidence and severity of radiation-induced xerostomia which is frequent documented as toxicity associated radiotherapy in head and neck cancer patients. However, there are few reports targeted at the clinical use of this procedure in the prevention and treatment of osteoradionecrosis in the mandibular. More evidence concerning amifostine's effectiveness in reducing the incidence and severity of osteoradionecrosis in the head and neck and the corresponding complications is needed in future research.

#### 3.2.3. Three-Dimensional (3-D) Printing

Three-dimensional (3-D) printing is a method of manufacturing in which materials, such as metal or plastic, are deposited upon one another in layers according to designs obtained from MRI, CT, or computer-aided design software to produce a 3-D object [83, 84]. Because it allows the manufacture of custom-designed constructs with higher complexity than conventional fabrication techniques, it is used in a large variety of medical applications including dentistry [85]. Many researchers have tried to reconstruct the head and neck's defects with this new approach. Three-dimensional (3-D) printing can be used in the fabrication of contour models of the reconstruction plates and dental implants [86]. Park et al. [13] used a 3D printer to fabricate resection guide and titanium mandibular implants. Dental implants were printed separately and manually installed on the titanium implant before surgery. Using the guide to resect the mandible affected by ORN, the titanium implants were inserted into the remaining mandible. Azuma et al. [87] compared 3-D printed contour models with conventional intraoperative bending for the bending of titanium plates when treating mandibular defects and revealed that 3-D printing shows better symmetry and mandibular angle compared to the conventional group. This 3D printing technology can greatly decrease surgical working time and provide a more precise design with less surgical bleeding, resulting in better outcomes [88]. However, 3-D technology also has some disadvantages, that upon opening to the resection site, the treatment plan often has to be revised on site in the face of unexpected pathology. Doctors are required to have enough experience to deal with an emergency situation during the operation.

#### 3.2.4. Progress in Tissue Engineering

As an alternative to the mandibular reconstruction by microanastomosed free flaps, preclinical tissular engineering is an appealing field in current research. A tissue engineering strategy using biphasic calcium phosphate (BCP) has been developed as an alternative to the standard reconstruction procedure [89]. Total bone marrow (TBM) associated with biphasic calcium phosphate (BCP) could enhance the irradiated bone's formation, being the most efficient mixture for the repair of irradiated bone currently [90]. The stromal vascular fraction (SVF) was reported to have the potential in bone reconstruction when used freshly digested and is easy and quick to obtain [91]. TBM in association with BCP appears to be the most efficient material for bone reconstruction after radiotherapy. Bone marrow cell extract (BMCE) from total bone marrow (TBM), containing intracellular factors, may be a contributor to the repair of irradiated bone through the paracrine effect [92]. However, before the application of tissue engineering to humans is taken into account, there are still multiple mechanisms and problems to be clarified.

#### 3.2.5. Sequence Therapy of the Mandibular Defect Caused by Osteoradionecrosis

Considering factors including clinical manifestations, imaging hints, oral and general health condition, a sequence therapy plan should be made based on the clinical severity. Treatment of ORN correlates with the severity of the disease, ranging from conservative therapy to extensive surgical resection and free flap reconstruction.

De Felice et al. [93] put forward that better ORN treatment is prevention, and treatment should be primarily nonsurgical. Conservative approaches such as removal of the irritants, improvement of oral hygiene, hyperbaric oxygen (HBO), ultrasound therapy (UST), and rational use of drugs including analgesics and anti-inflammatory drugs, antibiotics and/or broad-spectrum antimicrobials, and triple medicative therapy (pentoxifylline, tocopherol, and clodronate) may be effective and sufficient in the early stages [94–99]. However, surgical resection should be reserved for when a surgical operation will be necessary when the disease progresses into an advanced and/or refractory ORN despite conservative measures [97].

He et al. [31] proposed the treatment algorithm based on the BS staging system (Figure 4). Conservative treatment such as hyperbaric oxygenation (HBO), pentoxifylline, et al. may be considered for stage 0 patients (no distinct changes or just osteolytic images on radiography). Fistulas of Stage I patients can be surgically resected due to the small size of the affected tissues. Segmental mandibulectomy will be considered for most stage II and stage III patients to clear necrotic and unhealthy bony tissues.

For bilateral late-stage patients, synchronous or sequential resection and reconstruction can be considered depending on different circumstances. Li et al. [100] reviewed 22 patients with bilateral late-stage mandibular osteoradionecrosis who had failed to respond to conservative treatments and received mandibular radical resection, and 4 methods (bone flap repair, bone flap plus soft tissue flap, soft tissue flap, and soft tissue flap plus titanium plate repair) were used according to the individualized plan for each patient depending on their own local and general condition. All 22 cases obtained good wound healing or acceptable aesthetic and functional results. Aggressive management including segmental mandibulectomy and free flap reconstruction, is encouraged as it allows complete resection of the diseased segment, so no relapse/activation, or morbidity from marginal mandibulectomy will be encountered [101].

## 4. Discussion

In this review, we summarized the main research progress on osteoradionecrosis and reconstruction treatment of an osteoradionecrosis-caused mandibular defect. ORN usually develops during the first 6–12 months after radiotherapy; however, the risk remains for life [102]. Berger and Symington [103] reported that patients may still develop symptoms associated with ORN even more than 30 years after the end of radiation therapy. Previous studies have shown that prior chemoradiotherapy, smoking status, diabetes, resection sites, smoking history, and types of hardware used may be predictive of complications in the short-term, and predictors of long-term complications included prior chemoradiotherapy, cancer diagnosis, and resection sites [104, 105].

The mandible is a site of susceptibility to ORN in the head and neck region because of its compact and dense nature [106]. Although not the focus of this review, prophylactic measures for ORN in the mandible deserve sufficient clinical attention. Risk factors for mandibular ORN include surgical trauma and dental extractions following the radiotherapy. What's more, radiation dose, general health conditions, host immunity and surgical method may all play their parts in the development of osteoradionecrosis. Therefore, prophylactic management such as preirradiation dental care, intensity-modulated radiotherapy (IMRT), and the use of radiation protectors before radiotherapy must be taken into consideration in the treatment of the head and neck diseases requiring radiation therapy.

When osteoradionecrosis in the mandible has already occurred, it is often in need of complex multidisciplinary management, and the therapeutic protocols may range from conservative management to radical surgical strategies. It is commonly accepted as a principle that the therapeutic choice for ORN should be based on the patient's own general condition. Conservative treatments such as ultrasound therapy and hyperbaric oxygen therapy are usually implemented in the early stages and can also be used as a supplement to surgical management to achieve a better therapeutic effect.

Ultrasound therapy (UST) can induce angiogenesis and improve blood flow, so a UST protocol has been proposed in the treatment and prevention of ORN. Wu et al. [107] observed that low-intensity ultrasound can increase microvessel density and accelerate the healing of bone tissues from osteoradionecrosis in dogs. Zhou et al. [108] reported that compared without UST therapy, the irradiated dogs treated with UST though showed a better healing via improving vascularity and bone quality, the incidence of ORN showed no difference. It might be more cost-effective compared to HBOT, but it can only be suggested as an experimental option for cases of clinical trials limited by insufficient supporting evidence.

Though it is reported that after the contraindications such as existing malignant neoplasia, nontreated pneumothorax, optic neuritis, emphysema, and active viral infections ruled out, hyperbaric oxygen therapy (HBOT) can promote healing of wounds and decrease recovery time by facilitating the transfer of oxygen to the tissues [98, 109], though there are still skeptics of it. By reviewing the medical records of 47 patients who suffered from ORN, Kadakia et al. [110] found that the difference in flap breakdown between patients receiving and not receiving HBOT revealed no significant difference in outcome. Epstein et al. [111] concluded that complete remission is only achieved in 15% patients treated solely with conservative measures. Jenwitheesuk et al. [112] reported that for patients with severe ORN treated with HBOT alone without surgery, the signs and symptoms including severity of pain, swelling, wound discharge, and wound size can be decreased while not being cured. The use of HBOT alone or delays in surgical intervention may lead to unfavorable sequelae such as incomplete healing. Kün-Darbois and Fauvel [113] also supported the theory that hyperbaric oxygen therapy cannot heal ORN alone; it should be used as an adjuvant therapy in combination with surgery. Admittedly, surgery combined with HBOT leads to better wound healing in ORN patients than those treated with HBOT alone, surgeries are eventually inevitable. In addition to the expensive expense caused by the HBOT, there should be deliberateness about taking this medical procedure into routine use before reliable guidelines concerning the clinical use of the HBOT based on sufficient evidence are proposed.

Although the use of microanastomosed free-flap of the ORN of the mandibular in clinical practice is increasing number, it could also bring some complications including: (1) short-term complications (<4 weeks after surgery) such as fistula and dehiscence of the mucosal suture line and neck infection and (2) long‐term complications (>4 weeks after surgery) including tissue defects, exposure of the titanium plate, and pathological fracture. Walia et al. [104] showed that 30.5% of the patients who underwent oromandibular reconstruction had early complications, and the most common ones were wound dehiscence (11.3%) and fistulas (9.40%). 30.1% of them have a long-term complication, and the most common one is plate exposure (26.7%). Swendseid et al. [105] believed that those who experienced an early complication were not predisposed to also developing a long-term complication; only 11% of those with early complications developed a second complication. For those who need postoperative radiotherapy, we should keep in mind the preservation of mandibular periosteum during the surgical management of head and neck cancers. Without violating the rules of tumour extirpation, adequate soft tissue coverage or muscle attachment should be kept intact for a well-nourished microenvironment surrounding the cortical plate. Within the radiation field, unrepairable teeth due to caries, periodontal disease, or root lesions should be extracted to avoid local bacterial infection [114]. Though being a promising alternative to the mandibular reconstruction, for the tissue engineering to be used clinically, there are still long way to go.

At last but not least, although ORN results from noninfective hypovascular and hypocellular, late-stage mandibular ORN patients are more susceptible to secondary necrosis, superficial contamination, or fistulas [115]. Though advanced approaches to the reconstruction of an ORN-caused mandibular defect have been emerging, a reasonable perioperative anti-inflammatory treatment plan is not only a necessity but a must.

## 5. Conclusion

Since the mechanism of the ORN is not clear yet, the most widely accepted theories for now are still Marx's hypoxic-hypocellular-hypovascular theory and Delanian's fibro-atrophic theory. Ferroptosis may serve as one of the new possibilities to explain the development of ORN. The principles of treatment for mandibular ORN may be different depending on different staging system. Microanastomosed free-flap in mandibular ORN in clinical practice is in an increasing number, of which the fibular flap, providing long pedicles and abundant bone, is the most commonly used one. The free iliac crest flaps, scapula flaps, and other flaps such as ALT flaps and submental flaps that show adaptability in special clinical conditions are also applied to the reconstruction of the ORN-caused mandibular defect. Though advanced methods to treat and then reconstruct the mandibular defect caused by ORN including the use of three-dimensional (3-D) printing, pentoxifylline, and amifostine, as well as flap reconstruction in the mandible are gradually progressing, a sequence therapy plan should be made depending on the different clinical severity.

## Data Availability

No data were used to support this study.
